# Endogenous PYY and NPY mediate tonic Y_1_- and Y_2_-mediated absorption in human and mouse colon

**DOI:** 10.1016/j.nut.2008.06.015

**Published:** 2008-09

**Authors:** Helen M. Cox

**Affiliations:** a King's College London, Wolfson Centre for Age-Related Diseases, Guy's Campus, London, United Kingdom

**Keywords:** Peptide YY, Neuropeptide Y, Y receptors, Mucosal ion secretion, Absorptive tone

## Abstract

**Objective:**

To establish the functional significance of endogenous peptide YY (PYY) and neuropeptide Y (NPY) as mediators of Y_1_ and Y_2_ absorptive tone in colonic mucosa.

**Methods:**

Functional studies utilized descending colon from adult mice (wild type [WT] and peptide nulls) and ex vivo human colonic tissue (from patients undergoing bowel resections) measuring changes in basal ion transport. Peak increases in ion transport to Y_1_ or Y_2_ antagonists (BIBO3304 300 nM; BIIE0246 1 μM) were pooled (mean ± SEM) and compared using Student's unpaired *t* test (*P* ≤ 0.05); some tissues received tetrodotoxin (TTX; 100 nM). PYY-positive L-cell numbers and NPY innervation were also compared.

**Results:**

Y_1_ and Y_2_ tones were present in human and WT mouse colon mucosa and only the latter was TTX sensitive. Y_1_ tone was unchanged in NPY^−/−^ but was ∼90% inhibited in PYY^−/−^ and abolished in PYYNPY^−/−^ colon mucosa. Y_2_ tone was reduced ∼50% in NPY^−/−^ and PYY^−/−^ tissues and was absent from PYYNPY^−/−^ colon. Residual Y_2_ and Y_1_ tones present in PYY^−/−^ mucosa were abolished by TTX. PYY ablation had no apparent effect on NPY innervation and PYY-positive cells were observed at the same frequency in NPY^−/−^ (56.7 ± 6.8 cells/section) and WT (55.0 ± 4.6 cells/section) colons. Double knockouts lacked PYY and NPY expression, but endocrine cells and enteric nerves were present with similar frequencies to those of WT mice.

**Conclusion:**

Endogenous PYY mediates Y_1_ absorptive tone that is epithelial in origin, whereas Y_2_ tone is a combination of PYY and NPY mediation.

## Introduction

Peptide YY (PYY) and neuropeptide Y (NPY) are potent antisecretory peptides. Healthy human subjects infused with either peptide exhibit prolonged inhibition of pre-stimulated electrolyte secretion [1,2]. Recent functional studies utilizing selective Y-receptor antagonists and preferred peptide agonists have shown that PYY, NPY, their C-terminal fragments, and pancreatic polypeptide (PP) exert their sustained antisecretory actions via specific Y receptors, (Y_1_, Y_2_, and Y_4_) and that in human and mouse colons the same three Y receptors are involved [3–6]. However, clinical studies identifying the Y-receptor types responsible for these antidiarrheal actions have not been reported to date. Studies with ex vivo human colon have shown the presence of significant levels of Y_1_- and Y_2_-mediated absorptive tones [4,5]. In addition, mouse colon (but not other rodent colons) exhibits Y_1_ and Y_2_ tone [5–7] and the similarity in pharmacology exhibited by this rodent model and human tissue provides an opportunity to investigate the cellular mechanisms of absorptive tone further. This report is based on an invited lecture to the 9th International NPY meeting (Okinawa, March 2008) and focuses on the local mucosal effects of endogenous PYY and NPY.

### Peptides: their locations and functions in the intestine

Peptide YY is expressed predominantly in colorectal endocrine L-cells [8,9] and is colocalized with proglucagon products, glicentin and glucagon-like peptide-1 (GLP-1) and GLP-2 [10]. These peptides are copackaged [10,11] and co-released when food reaches the duodenum. PYY is also found in pancreatic endocrine F-cells, often with PP. In the enteric nervous system, NPY is present in ∼50% of submucous plexus neurons, the majority of which innervate the mucosa and target the lamina propria of most species, including rat, mouse, and human colon [12,13]. Within the submucous plexus, NPY is most frequently present in secretomotor nerves and is colocalized with the inhibitory neurotransmitter, vasoactive intestinal polypeptide [12,14]. PP, in contrast, is present in a sparse population of endocrine cells scattered along the length of the intestine in most species [9,10], its primary source being the pancreatic F-cells.

Dietary fat, bile salts, carbohydrates, and proteins can stimulate PYY release but to different degrees and with different rates (for review, see Onaga et al. [15]). PYY release is also regulated by, and in turn regulates, vagal nerve activity [15] and the hormone is a major mediator of ileal and colonic brakes, mechanisms that ultimately slow gastric emptying and promote digestive activities to increase nutrient absorption [16–18]. In the circulation ∼40% of released PYY is converted to PYY(3–36) [19,20] and the consequence of this conversion is to amplify Y_2_-mediated mechanisms at the expense of Y_1_ (and Y_4_) responses, because the long fragments have a low affinity for the latter receptors. This hydrolysis occurs via the serine protease, dipeptidyl peptidase-4 (DPP4; EC 3.4.14.5), which cleaves aminoterminal dipeptides from NPY with a higher maximum activity than from PYY [20]. The switching of receptor activities is likely to be important in modulating digestive behavior and in initiating satiety consequent to postprandial increases in plasma PYY and PYY(3–36). DPP4 inhibitors are clinically important because they also prolong the half-life of incretin hormones, GLP-1 and GLP-2 [21], both of which lower blood glucose in a glucose-dependent manner. The inhibitors' potential as new treatments for type 2 diabetes is proven [22] but a proportion of their effects (i.e., promoting satiety and reducing food intake and subsequently body weight) are likely to be mediated by increased half-lives of unrelated peptides, e.g., PYY or NPY, whose prolonged stability (together with other DPP4 substrates [23]) may result in acute intestinal side effects.

### Y receptors: their locations and implications for intestinal function

Exogenous PYY, NPY (their fragments), and PP are potent, broad-spectrum inhibitors of electrolyte secretion in human [2,4] and mouse [3,5] intestines and functional studies utilizing genetically modified mice lacking a Y receptor (Y_1_^−/−^, Y_2_^−/−^, or Y_4_^−/−^) combined with selective Y_1_ [24] or Y_2_ [25] antagonists and neurotoxin-treatment have shown that Y_1_ and Y_4_ are predominantly epithelial, whereas Y_2_ receptors are neuronal (Y_5_ (ant)agonists have no effect) [3–5]. Y_1_^−/−^ mouse tissues are predictably insensitive to Y_1_ agonists and selectively lack Y_1_ absorptive tone, whereas Y_2_ tone and Y_2_ agonist sensitivity are unaltered [7]. Chemical depolarization of intrinsic submucous neurons (with veratridine) causes sustained epithelial responses that are not altered by Y_1_ antagonism (BIBO3304) or ablation (i.e., in Y_1_^−/−^ tissue [7]). Predictably Y_2_^−/−^ mucosa is Y_2_ agonist–insensitive and lacking in Y_2_ absorptive tone but Y_1_ tone is unchanged [5]. Neurogenic mucosal responses in wild-type (WT) colon are amplified by Y_2_ antagonist pretreatment and are significantly increased in Y_2_^−/−^ colon [5]. Irrespective of these differences in cellular localization, Y-receptor activation leads consistently to prolonged antisecretory effects.

Immunohistochemical studies have revealed extensive Y_1_ labeling on epithelial basolateral membranes and discrete labeling of intrinsic neurons in the lamina propria of pediatric and adult human colon [13,26]. PYY-positive endocrine cells are often surrounded by Y_1_-immunoreactive epithelia and nerve fibers but are themselves Y_1_-negative [26]; thus a paracrine rather than an autocrine action for PYY is likely to be significant in human colon. In the rat intestine numerous Y_1_-positive cell bodies have been observed in myenteric but not in submucous ganglia [27], implicating a neuromodulatory role in smooth muscle activity in this species. In rat jejunum and colon Y_2_ expression occurs in epithelial and muscle layers [28] but as yet no immunohistochemical Y_2_-receptor localization in the intestine has been described. Notably, Y_2_ or Y_1_ absorptive tone is absent in rat gastrointestinal tissues [29]. In human and mouse colon mucosae, however, there is significant endogenous Y_2_ tone, which appears to be neurogenic [3,5] and is therefore more likely to be NPY rather than PYY mediated [7].

Despite growing pharmacological evidence, a better understanding of the endogenous mechanisms underpinning tonic absorption is a prerequisite if Y receptors are to be considered potential targets for treating malabsorption. Therefore, we set out to establish whether endogenous PYY was responsible for Y_1_ absorptive tone and NPY-mediated Y_2_ tone, making use of single (PYY^−/−^ and NPY^−/−^) and double (NPYPYY^−/−^) knockout mice in conjunction with human colonic tissue.

## Materials and methods

BIBO3304 and BIIE0246 were gifts from Boehringer-Ingelheim Pharma KG (Biberach an der Riss, Germany) and stocks in 10% dimethyl sulfoxide were stored at −20°C. Peptides were from Bachem Laboratories Inc. (St. Helens, United Kingdom). The DPP4 inhibitor, compound 3, was a gift from Dr. R. Roy (Merck Inc., Rahway, NJ, USA). Anti-PYY (from Dr. E. Ekblad, University of Lund, Lund, Sweden), anti–C-terminal peptide or NPY and anti-NPY antibodies (Affiniti Research Products Ltd., Exeter, United Kingdom), and goat anti-rabbit fluorescein isothiocyanate– or tetramethylrhodamine isothiocyanate–conjugated secondary antibodies (BIOMOL International, Exeter, United Kingdom) were used. All other compounds were of analytical grade (Sigma-Aldrich, Poole, United Kingdom).

### Measurement of ion transport and Y_1_ and Y_2_ absorptive tone in isolated mucosal preparations

Colon mucosa from clinical specimens obtained from consenting patients undergoing bowel resection surgery (three men and one woman; Table 1) or from WT and knockout mice of different genotypes (NPY^−/−^, PYY^−/−^ [30] and double knockouts, 16–24 wk old, either gender, with a mixed C57BL/6–129/SvJ background, fed standard chow) were prepared by removing overlying smooth muscle and voltage-clamped at 0 mV in Ussing chambers as described previously [3,4]. Vectorial ion transport (I_sc_) was measured continuously as microamps per square centimeter and all additions were basolateral. Once stable basal I_sc_ levels were achieved, mucosae were pretreated with vehicle, the DPP4 inhibitor (1 μM of compound 3, [22]) or tetrodotoxin (TTX; 100 nM). Treatment periods were 20–30 min before the addition of the Y_1_-receptor antagonist BIBO3304 (300 nM [24]), the inactive enantiomer of BIBP3226, BIBP3435 (1 μM, 3435), or the Y_2_ selective antagonist BIIE0246 (1 μM [25]). The maximum rise in I_sc_ (15–25 min) after each Y antagonist was pooled and compared with controls.

### Immunohistochemistry

Lengths (2–3 cm) of mouse descending colon were washed in KH buffer and immersed in paraformaldehyde (4%) for 24 h, washed well in phosphate buffered saline (PBS), cryoprotected in 30% sucrose in PBS for 48 h before being embedded in OCT (VWR International, Lutterworth, UK), and stored at −80°C. Sections (15 μm) were cut, rehydrated in PBS, and blocked in 10% normal goat serum in PBS for 2 h before incubating overnight in polyclonal anti-PYY antibody (1:1000) to visualize PYY-containing endocrine cells or in chromogranin A (1:400) to label all endocrine cells. Longer incubation times (3–4 d) were used to enable anti-NPY labeling (1:400) of NPY-containing neurons or protein gene product (PGP)9.5 (1:400) labeling of all enteric neurons. Primary antibodies were visualized with goat anti-rabbit F(ab′)_2_ secondary antibodies conjugated to fluorescein isothiocyanate or tetramethylrhodamine isothiocyanate (used at 1:200 for 2 h at room temperature; Chemicon, Harrow, UK). The sections were washed in PBS, mounted in Fluorosave (Calbiochem, Nottingham, UK), and viewed with a Provis microscope fitted with appropriate filters and Axiovision software, and the numbers of fluorescent endocrine cells were counted and innervation compared between genotypes.

### Data analyses

Maximal changes in I_sc_ at 15 or 25 min are expressed throughout as mean ± SEM from a minimum of three experiments. Single comparisons between data groups were performed using Student's unpaired *t* test, whereas multiple comparisons used one-way analysis of variance with Dunnett's post-test with *P* ≤ 0.05 considered statistically significantly different.

## Results

**Fig. 1:**
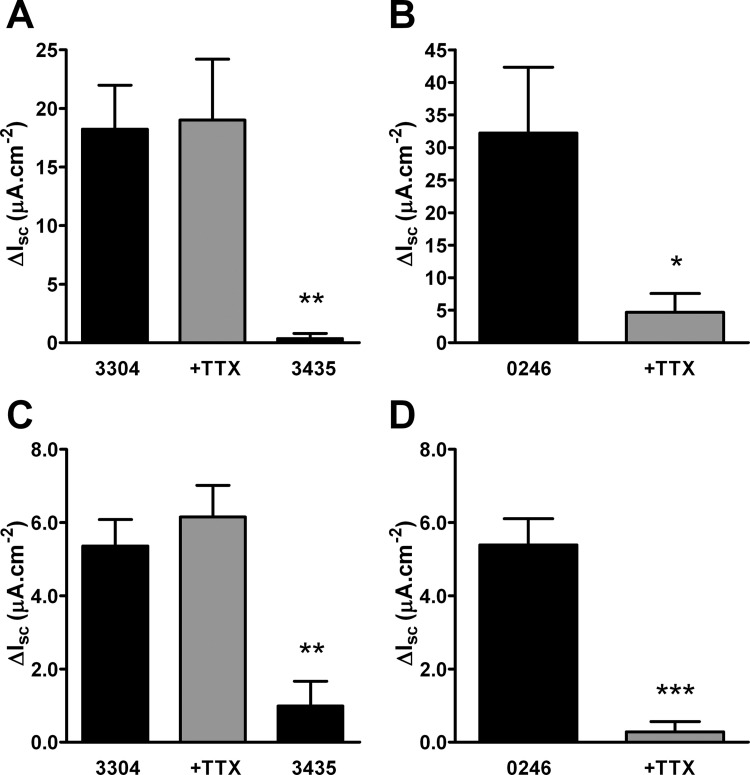
Y_1_ (3304) and Y_2_ (0246) antagonists reveal absorptive tone but 3435 (an inactive Y_1_ isomer) was ineffective. Y_1_ and Y_2_ antagonism raised I_sc_ in human (A, B) and wild-type (C, D) mouse colon mucosa, respectively. Y_1_ tone in both tissues was insensitive to TTX (+TTX, 100 nM; A, C), whereas Y_2_ tone was significantly reduced by TTX pretreatment of both mucosae (B, D). Asterisks indicate statistical differences between control and experimental data groups (*P ≤ 0.05, **P ≤ 0.01, ***P ≤ 0.001) and bars represent mean ± SEM from 3–10 observations. ΔI_sc_, change in short-circuit current; TTX, tetrodotoxin; 0246, BIIE0246; 3304, BIBO3304; 3435, BIBP3435.

Table 1 presents the basal resistances and I_sc_ levels for human and murine colon mucosae. Values were similar to those published previously for human and WT mouse mucosae [5,6] and basal levels of I_sc_ and TTX-sensitive I_sc_ in NPY^−/−^ colon specifically were significantly higher than those of WT tissue. The competitive Y_1_ antagonist, BIBO3304, caused sustained elevations in I_sc_ that were maximal at 15 min in WT mouse and human colon mucosa and neither of these effects was sensitive to TTX pretreatment (Fig. 1A,C). The inactive Y_1_ antagonist enantiomer, BIBP3435, had no effect per se (*P* ≤ 0.01 in both tissues). Blockade of Y_2_-mediated absorption (with Y_2_ antagonist BIIE0246) also increased basal I_sc_ levels that were virtually abolished by the neurotoxin TTX (Fig. 1B,D). This indicates that Y_2_ tone is predominantly neuronal in contrast to Y_1_ absorptive tone that is non-neuronal in both colonic tissues.

Because NPY is a better substrate for DPP4, Y_2_ tone was predicted to be amplified by a selective DPP4 inhibitor. Whereas Y_1_ tone was unaffected in mouse or human mucosa (data not shown), the same pretreatment with compound 3 significantly augmented Y_2_ tone at 25 min in human mucosa (control [*n* = 4] 9.6 ± 4.7 μA/cm^2^ versus compound 3 pretreatment [*n* = 4] 29.5 ± 5.9 μA/cm^2^, *P* ≤ 0.05) and at 15 min after BIIE0246 addition to mouse mucosa (controls [*n* = 8] 8.7 ± 2.3 μA/cm^2^ versus pretreatment [*n* = 8] 17.1 ± 2.6 μA/cm^2^, *P* ≤ 0.05).

**Fig. 2:**
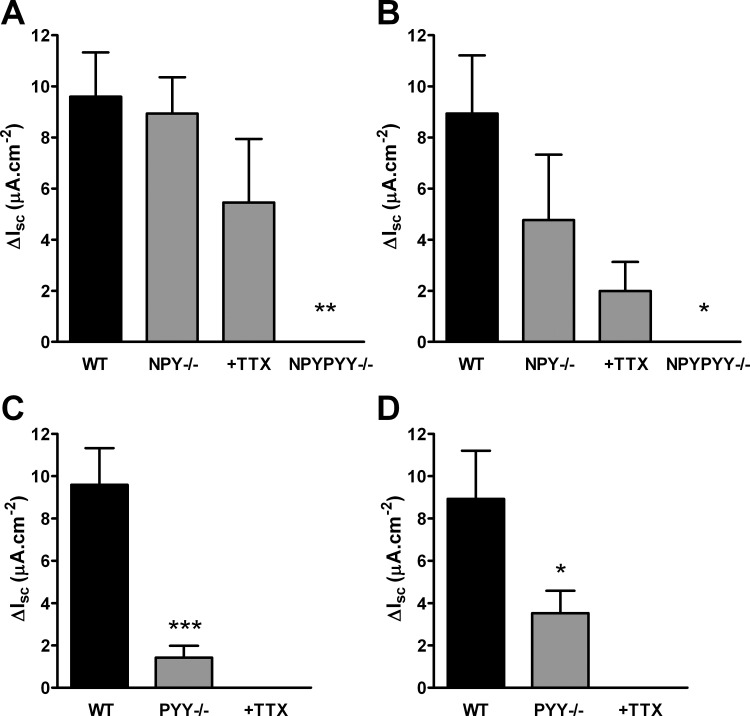
Effects of individual NPY or PYY ablation (NPY^−/−^, PYY^−/−^) and double knockout (NPYPYY^−/−^) on Y_1_ and Y_2_ tone in mouse colon mucosa. (A) WT Y_1_ tone was similar to that of NPY^−/−^ with or without TTX but was absent from NPYPYY^−/−^ tissues (**P ≤ 0.01). (B) Y_2_ tone was partially reduced in NPY^−/−^ tissue with or without TTX (not significantly) and abolished in NPYPYY^−/−^ preparations (*P ≤ 0.05). (C) Y_1_ tone was reduced in PYY^−/−^ colon (***P ≤ 0.001) and residual Y_1_ tone was abolished by neuronal blockade with TTX. (D) Y_2_ tone was partially inhibited in PYY^−/−^ mucosae (*P ≤ 0.05) and abolished by TTX. Bars represent mean ± SEM from 3–7 observations. ΔI_sc_, change in short-circuit current; NPY^−/−^, neuropeptide Y single knockout; NPYPYY^−/−^, peptide YY/neuropeptide Y double knockout; PYY^−/−^, peptide YY single knockout; TTX, tetrodotoxin; WT, wild-type.

To establish the relative contributions of endogenous NPY and PYY toward each Y-receptor–mediated tone, we monitored the effects of Y_1_ or Y_2_ antagonists in colon mucosa from single knockout mice (NPY^−/−^ or PYY^−/−^) and the double-null mice (NPYPYY^−/−^). Comparison of the maximal increases in I_sc_ 15 min after antagonist additions in WT versus null mucosa showed that NPY^−/−^ colon exhibited normal Y_1_ tone (that was not TTX sensitive; Fig. 2A), whereas Y_2_ tone was partially (but not significantly) reduced by NPY ablation (Fig. 2B). TTX pretreatment of NPY^−/−^ tissue did not further inhibit Y_1_ or Y_2_ residual tone (Fig. 2A,B). In marked contrast, PYY^−/−^ mucosa was markedly less sensitive than WT colon to Y_1_ (Fig. 2C) and Y_2_ (Fig. 2D) antagonism and blocking neuronal activity in this tissue abolished these residual increases in I_sc_. NPYPYY^−/−^ tissues were insensitive to both Y antagonists (Fig. 2A,B).

The NPYPYY^−/−^ colon lacked PYY endocrine cells and NPY-containing neurons in both intramural plexi, although chromogranin A–stained endocrine cells and PGP9.5-positive neurons were present (data not shown). The PYY^−/−^ colon exhibited no obvious alteration in the pattern of intramural NPY innervation, but PYY-positive cells were absent. The NPY^−/−^ colon exhibited similar numbers of PYY-labeled endocrine cells (56.7 ± 6.8 cells/section) compared with WT mucosa (55.0 ± 4.6 cells/section) and the morphology of WT and null tissues was similar.

## Discussion

Y_1_ tone is clearly stereospecific and not mediated by TTX-sensitive enteric neurons in human and mouse colon (Fig. 1A,C). In contrast, Y_2_ tone is predominantly neurogenic (Fig. 1B,D), as described previously in normal human colon and WT mouse mucosa [4,5]. The link between NPY and Y_2_ tone was initially revealed in ex vivo studies showing that NPY^−/−^ and Y_2_^−/−^ tissues lost Y_2_ tone and were indistinguishable in this regard [7]. The present study included NPY^−/−^ mice from a source different from that used previously [7] but with a similar mixed genetic background (C57BL/6–129/SvJ). Apart from an elevation in basal I_sc_ and TTX sensitivity in NPY^−/−^ compared with WT values (Table 1), there were no differences in electrophysiological parameters between genotypes. Loss of inhibitory NPY could be partly responsible for the increase in basal I_sc_ levels if tonic activation of intrinsic submucous nerves is contributing to basal I_sc_. The increased effect of TTX on basal I_sc_ in NPY^−/−^ compared with WT values would also confirm this is the case.

The selective amplification of Y_2_ tone by DPP4 inhibition also indicates a significant tonic inhibition for endogenous NPY in human and mouse colon. Prolonging NPY's half-life would result in potentiation of neurogenic responses and, because Y_2_ effects are primarily neuronal, they were increased, whereas the antisecretory actions of PYY at epithelial Y_1_ receptors were not significantly altered. Peptide levels before and after DPP4 inhibition will be measured to confirm the relative stability of NPY over PYY, particularly at times when Y_2_ (but not Y_1_) tone is significantly amplified.

**Fig. 3:**
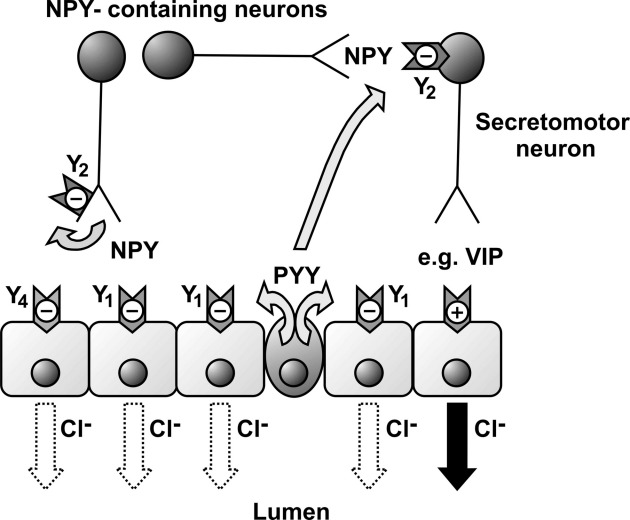
Schematic diagram showing the intramural sites of action of endogenous PYY and NPY in normal mouse and human colon mucosa. Direct activation of epithelial Y_1_ receptors by PYY (and NPY) will inhibit epithelial anion (Cl^−^) secretion. NPY released from submucosal secretomotor neurons can auto-inhibit its release (lefthand side, a Y_2_-mediated effect) and also, when released from interneurons, can inhibit (again via Y_2_ receptors) secretomotor (e.g., VIP-ergic) neurons. Endocrine PYY can co-activate neuronal Y_2_ receptors and predominant epithelial Y_1_ receptors, and both mechanisms result in sustained inhibition of epithelial Cl^−^ secretion. The NANC neurotransmitter in the final secretomotor neuron (righthand side) has yet to be positively identified but is likely to be VIP, which in turn stimulates prolonged epithelial cyclic adenosine monophosphate–dependent Cl^−^ secretion that can be inhibited by Y_1_ (or Y_4_) receptor activation. NPY, neuropeptide Y; PYY, peptide YY; VIP, vasoactive intestinal polypeptide.

Knockout of individual peptides revealed clear differences in functional losses in the absence of any changes in peptide distribution. The NPY^−/−^ colon exhibited normal levels of Y_1_ tone that were not significantly altered by neuronal blockade, indicating a direct epithelial mechanism of PYY action (Fig. 2A,B). This agrees with previous studies of antisecretory responses activated by exogenous analogs [5,6] (Cox et al., unpublished observations). PYY ablation conversely reduced Y_1_ tone by ∼90% (Fig. 2C), whereas Y_2_ tone was partially inhibited (Fig. 2D) and the remaining residual responses were abolished by TTX and by inference must be NPY mediated. As predicted, double knockouts lacked Y_1_ and Y_2_ tonic absorption (Fig. 2A,B). We conclude that a combination of the two peptides are responsible for Y_2_ tonic absorption and that neuronal and epithelial mechanisms underpin this tonic effect, as indicated by the neurotoxin TTX's ability to partially inhibit antagonist responses in NPY^−/−^ (Fig. 2B) and abolish remaining Y_2_ tone in the PYY^−/−^ colon (Fig. 2D). In contrast, NPY has a minor role as a mediator of Y_1_ tone (Fig. 2C). The location of Y_1_ receptors in basolateral poles of epithelia surrounding PYY-positive endocrine cells in human colon [26] fits well with the functional PYY–Y_1_ interactions observed in this tissue and in mouse colon (as shown in Fig. 3). In these tissues we have no evidence for submucous plexus or mucosal neuron Y_1_ expression and this agrees with the more limited distribution of Y_1_-receptor immunoreactivity noted by Matsuda et al. [27]. They observed only Y_1_ labeling of myenteric nerves (removed with smooth muscle from our preparations), a few endocrine-like cells, and specific larger blood vessels.

The selective inhibition of Y_2_ responses by TTX indicate the absence of Y_2_ receptors in human and mouse colonic epithelia, in contrast to previous studies of rat jejunum, where functional studies [29] and polymerase chain reaction–based detection of Y_2_ mRNA [28] showed epithelial Y_2_ expression. We propose that activation of presynaptic Y_2_ receptors by NPY and PYY inhibits secretomotor nerves (most probably vasoactive intestinal polypeptide–ergic) and provides a mechanism by which Y_2_ antagonism elevates basal I_sc_ and causes hypersecretion (Fig. 3) in human and mouse colon. NPY released from other non-adrenergic, non-cholinergic secretomotor nerves could auto-inhibit NPY release by Y_2_ receptor activation and local NPY, NPY(3–36), PYY, or PYY(3–36) could act on these neuronal Y_2_ receptors to modulate ongoing mucosal electrolyte secretion.

Ultimately, whatever the luminal or neural (vagal) stimulus, released PYY will rapidly activate several local (paracrine) targets, primary among them, the epithelium. In addition to its hormonal effects that occur within minutes to hours, PYY can also exert longer-term responses to alter epithelial adhesion, differentiation, and increasing cell migration [31], potentially protecting against stimuli that cause mucosal erosion. The same PYY^−/−^ mice that we have used have been shown to exhibit a more prominent female development of mild late-onset obesity with a high-fat diet [30] and this outcome was not dissimilar to the age-related weight gained by both genders of another PYY^−/−^ recently described [32]. Whether hyperinsulinemia contributes to this phenotype is not agreed but tonic inhibition afforded by PYY acting on Y_1_ receptors in pancreatic islets combined with Y_2_ inhibition of vagal output have been proposed [30] as coincident contributing factors. It is interesting to note that PYY and NPY are responsible for Y_1_- and Y_2_-mediated tonic inhibition in central and peripheral targets including the intestinal tract, where I have uncovered significant tonic activity using selective Y_1_ and Y_2_ antagonists.

In conclusion, PYY and NPY exert common final antisecretory actions but mediated by different pathways involving two Y receptors (Y_1_ and Y_2_) with distinct distributions. The PYY-synthesizing L-cells of the large bowel play a pivotal role, not only in the regulation of satiety and ileal brake but also in response to different luminal cues, e.g., fatty acid chain length [33], resulting in predominant Y_1_ absorptive responses. NPY exerts its inhibitory effects predominantly through submucous neuronal innervation of the mucosa to bring about indirect Y_2_-mediated absorption and both tonically active mechanisms are present in human and mouse colon mucosa.

## Figures and Tables

**Table 1:** Age and gender comparisons with basal resistance and I_sc_ levels and the effect of neuronal blockade by TTX in mucosal preparations from human and murine colon⁎

	Age	No./gender	Resistance (Ω, cm^2^)	Basal I_sc_ (μA/cm^2^)	TTX (μA/cm^2^)
Humans	71.3 ± 7.0 y (4)	3/M, 1/F	75.5 ± 4.9 (21)	72.0 ± 7.6 (21)	−30.5 ± 5.3 (7)
Mice					
WT	18.5 ± 1.2 wk (17)	15/M, 2/F	29.3 ± 1.8 (52)	55.3 ± 5.6 (52)	−3.6 ± 1.3 (17)
NPY^−/−^	24.1 ± 1.4 wk (9)	5/M, 4/F	28.2 ± 2.7 (18)	82.6 ± 8.6 (18)†	−9.4 ± 1.8 (7)†
PYY^−/−^	18.3 ± 3.3 wk (7)	7/M	37.3 ± 3.0 (20)	73.9 ± 9.8 (20)	−8.6 ± 2.8 (6)
NPYPYY^−/−^	16.3 ± 0.5 wk (4)	4/F	24.8 ± 3.1 (8)	51.9 ± 15.1 (8)	ND

F, female; I_sc_, short-circuit current; M, male; ND, not determined; NPY^−/−^, neuropeptide Y single knockout; NPYPYY^−/−^, peptide YY/neuropeptide Y double knockout; PYY^−/−^, peptide YY single knockout; TTX, tetrodotoxin; WT, wild-type.

⁎ Values are means ± SEMs (numbers of observations).

† *P* < 0.05 compared with WT controls.
